# The Box—eHealth in the Outpatient Clinic Follow-up of Patients With Acute Myocardial Infarction: Cost-Utility Analysis

**DOI:** 10.2196/30236

**Published:** 2022-04-25

**Authors:** Roderick Willem Treskes, M Elske van den Akker-van Marle, Louise van Winden, Nicole van Keulen, Enno Tjeerd van der Velde, Saskia Beeres, Douwe Atsma, Martin Jan Schalij

**Affiliations:** 1 Department of Cardiology, Leiden University Medical Center Leiden Netherlands

**Keywords:** smart technology, myocardial infarction, cost-utility, outpatients, cost-effectiveness, eHealth, remote monitoring, cost of care, quality of life

## Abstract

**Background:**

Smartphone compatible wearables have been released on the consumers market, enabling remote monitoring. Remote monitoring is often named as a tool to reduce the cost of care.

**Objective:**

The primary purpose of this paper is to describe a cost-utility analysis of an eHealth intervention compared to regular follow-up in patients with acute myocardial infarction (AMI).

**Methods:**

In this trial, of which clinical results have been published previously, patients with an AMI were randomized in a 1:1 fashion between an eHealth intervention and regular follow-up. The remote monitoring intervention consisted of a blood pressure monitor, weight scale, electrocardiogram device, and step counter. Furthermore, two in-office outpatient clinic visits were replaced by e-visits. The control group received regular care. The differences in mean costs and quality of life per patient between both groups during one-year follow-up were calculated.

**Results:**

Mean costs per patient were €2417±2043 (US $2657±2246) for the intervention and €2888±2961 (US $3175±3255) for the control group. This yielded a cost reduction of €471 (US $518) per patient. This difference was not statistically significant (95% CI –€275 to €1217; *P*=.22, US $–302 to $1338). The average quality-adjusted life years in the first year of follow-up was 0.74 for the intervention group and 0.69 for the control (difference –0.05, 95% CI –0.09 to –0.01; *P*=.01).

**Conclusions:**

eHealth in the outpatient clinic setting for patients who suffered from AMI is likely to be cost-effective compared to regular follow-up. Further research should be done to corroborate these findings in other patient populations and different care settings.

**Trial Registration:**

ClinicalTrials.gov NCT02976376; https://clinicaltrials.gov/ct2/show/NCT02976376

**International Registered Report Identifier (IRRID):**

RR2-10.2196/resprot.8038

## Introduction

eHealth, broadly speaking, the delivery of medicine using information technology, has been suggested as a cost-saving tool to deliver health care [1,2]. It can be delivered using personal computers, mobile phones, or tablets. One advantage of delivering health care through these mobile devices is that it uses an already existing infrastructure. The vast majority of the western world population has internet access or possesses a smartphone. Recent statistics showed that 92% of the Dutch population (aged ≥12 years) uses the internet, and 89% of the population owns a smartphone [3].

Mobile technology might be cheaper than conventional health care technology. Furthermore, an eHealth intervention can be delivered to more patients at the same time using information technology [4]. This also allows health care delivery in low- and middle-income countries. In addition, if it decreases the costs of health care delivery in high-income countries, it may increase equality.

Accordingly, smartphone-compatible devices might be clinically effective and cost-saving tools to deliver health care to acute myocardial infarction (AMI) patients. In The Box trial, a trial randomizing 200 patients to either an eHealth intervention or regular follow-up, it was found that there was no difference in clinical endpoints. A cost-effectiveness analysis of this trial was not included [5]. It is, therefore, the primary purpose of this study to describe a cost-utility analysis of an eHealth intervention (The Box) compared to regular follow-up in the outpatient care setting of patients who have been treated for AMI with primary percutaneous coronary intervention (PCI), with or without ST elevation, using data from The Box trial.

## Methods

### Overview

“The Box” was a single-center open-label randomized controlled trial (RCT) conducted at the Department of Cardiology of the Leiden University Medical Center (LUMC) in Leiden, the Netherlands, between May 2016 and December 2018 (NCT02976376) [5]. The current paper describes a trial-based cost-utility analysis of the intervention.

### Intervention

Details about the trial protocol and the results of the clinical trial have been published previously [5,6]. In brief, patients who were admitted to the cardiac care unit (CCU) of the LUMC with an AMI, as defined by European Society of Cardiology (ESC) guidelines [7,8], were approached for participation. Both patients with ST-elevation myocardial infarction (STEMI) and patients presenting with non-ST-elevation myocardial infarction (NSTEMI) were eligible for participation. Therefore, according to the ESC guidelines, every patient admitted to the CCU, with symptoms of AMI, elevated troponin levels, and a more than 90% occlusion on coronary angiography, which was treated with primary PCI ≤48 hours after onset of symptoms, was considered for participation [9]. Patients were excluded if they were ≤18 years old, pregnant, unwilling or unable to sign the informed consent form, included in another RCT, or unable to communicate in English or Dutch at a sufficient level. After inclusion, patients were randomized to either the intervention group or the control group. When randomized to the control group, patients were followed-up according to the department’s AMI follow-up protocol (called MISSION! protocol) [10]. Patients visited the outpatient clinic 1 month, 3 months, 6 months, and 12 months after they were treated for AMI. At each visit, a 12-lead electrocardiogram (ECG) was obtained, and blood pressure (BP) was measured by a nurse practitioner (NP) with ample training using a handheld sphygmomanometer. At 3 months, a stress echocardiogram was done, and a 24 Holter monitor was attached to the patient. At 6 months, a transthoracic echocardiogram (TTE) was done, and a 24 Holter monitor was performed. At 12 months, a TTE was done. Patients were not monitored in between outpatient clinic visits. When randomized to the intervention group, patients received a box containing a smartphone-compatible weight scale, a BP monitor, a step counter (all three by Nokia Health, Nokia), and an ECG device (Kardia, AliveCor Inc). Patients were asked to record their weight, BP, and ECG once daily and to record their steps taken continuously. Data were automatically transferred from the patient’s smartphone to the department’s dedicated hospital information system (EPD-Vision), and data were checked multiple times per week. In case of possible abnormalities (high BP, possible arrhythmias, or a sudden increase or decrease in weight), patients were contacted by a doctor or NP, and the therapeutic regimen could eventually be adjusted. Furthermore, the outpatient clinic visits 1 month and 6 months after AMI were replaced by an e-visit, in which the patient contacted the hospital via a secured video connection (Starleaf Breeze, Starleaf). The ECG at the 1- and 6-month outpatient clinic visit, as well as the TTE and the 24 hour Holter monitor at the 6-month outpatient clinic visit, were not performed in the intervention group. In case of technical difficulties, patients could contact a project dedicated PhD Student for technical support. This technical support was primarily delivered via telephone or a secured video connection. If problems could not be solved, a computer expert would visit the patient at their home.

### Dutch Health Care System

Detailed information (in English) on the Dutch health care system is published elsewhere [11]. In brief, the law that covers the payment for hospital care is called the Health Care Insurance Act (in Dutch: “Zorgverzekeringswet”). The system combines aspects of private and public insurance. Health care insurers are private companies that are not-for-profit. The health care insurance act demands that healthcare insurers accept all customers, regardless of their health care condition. Insurers are furthermore forbidden to charge different premiums for the same package. Finally, insurers are obliged to make health care that is part of the government decided basic health package available to all customers. All residents of the Netherlands are obliged to take health insurance. Health care insurers negotiate prices and volumes of care with hospitals, focusing on affordability and quality of care. Hospitals either employ doctors or doctors are working on a fee-for-service basis. Patients pay a deductible of €385 (US $423) and a fixed premium price per month [11].

In this trial, costs of The Box are covered by the Department of Cardiology of the LUMC. The diagnosis-related group of a PCI in AMI is more than €385 (US $423). As such, patients had to pay their deductible. Therefore, as premium prices are fixed and do not depend on the amount of health care consumed, there was no difference in the amount of money patients had to pay for health care between patients who participated in the intervention group or the control group.

### Trial Based Analysis

The trial-based cost-utility analysis was performed from a department of cardiology’s perspective with a time horizon of 1 year. All costs are reported in 2020 euros. The general Dutch consumer price index was used to convert costs to 2020 price levels [12]. The analysis was performed on a modified intention-to-treat population. To create such a population, 12 (12%)patients in the intervention group and 8 (8%) patients in the control group were included in the trial but dropped out within two weeks due to various reasons and, therefore, not following the protocol as planned, were excluded from the analysis. For the base-case analysis, only health care consumption at the cardiology department (defined as the cardiac care unit, emergency room, ward, and outpatient clinic) was taken into consideration. The intervention primarily intervenes with follow-up of cardiac care and targets some specific risk factors for cardiovascular disease. It is therefore assumed there is no causal relationship between our intervention and health care utilization of other departments or outside the hospital. These costs are therefore not taken into account. All calculations were done in Excel and SPSS (version 23.0, IBM Corp) and IBM SPSS Statistics for Windows (version 22; IBM Corp).

### Cost Calculations

#### Costs of The Box

Costs for the intervention (The Box) and the technical support of The Box were copied from bills received by the department. Costs for procedures performed as part of the study protocol (stress echocardiogram, transthoracic echocardiogram, 24-Hour Holter monitor, digital outpatient clinic visit, and in-office outpatient clinic visit) were at LUMC prices. Extra consultations for adjustment of the therapeutic regimen as a consequence of irregularities in Box data were included in the intervention group. A consult of 10 minutes was multiplied by the hourly wage of a nurse practitioner. All cost prices are given in Table 1.

**Table 1:** Costs of The Box, follow-up, and major adverse cardiac events.

Item			Price		Source
The Box (ECG^a^ monitor, blood pressure monitor, weight scale, cardboard box, manual)			€318^b^		Bills
**Follow-up**					
	Stress echo	€542		LUMC^c^	
	Echo (outpatient clinic)	€117		LUMC	
	24-hour Holter monitor	€152		LUMC	
	E-visit outpatient clinic visit	€44		LUMC	
	Normal outpatient clinic visits	€96		LUMC	
Technical support			€1758		Bills
Contact NP^d^ due to Box measurements (per contact)			€4		Dutch costing guidelines
Coronary angiogram			€2037		NZA^e^
Revascularization (elective), 1-vessel disease (1VD), with admission			€5999		NZA
Revascularization (elective), multivessel disease (MVD), with admission			€6428		NZA
Admission, unspecified (price per night)			€684		Dutch costing guidelines
In hospital technical support (including training of patients to use devices and checking of data)			€15,367		UMC^f^ gross salary of 0.5 FTE^g^

^a^ECG: echocardiogram.

^b^A currency exchange rate of €1=US $1.0994 is applicable.

^c^LUMC: Leiden University Medical Center.

^d^NP: nurse practitioner.

^e^NZA: Nederlandse Zorgautoriteit (Dutch Healthcare Authority).

^f^UMC: University Medical Center.

^g^FTE: full-time equivalent.

#### Follow-up Hospitalization Costs

The following events were taken into account: during follow-up, cardiac care utilization due to nonfatal adverse cardiac events (defined as any hospital visit for myocardial infarction, elective revascularization, arrhythmia, or heart failure) was noted. The following events were defined: coronary angiography without intervention, revascularization (elective), recurrent STEMI, recurrent non-ST elevation acute coronary syndrome, acute heart failure, and hospital admission to the cardiac care unit or cardiology ward for other reasons than the above. Costs for revascularization were taken from the Dutch Health care Authority (in Dutch: “Nederlandse Zorgautoriteit”; NZA) costing lists [13]. These lists distinguish four types of elective revascularization: single vessel revascularization with and without an overnight stay in the hospital and multivessel revascularization with or without an overnight stay in the hospital. Costs for revascularization with an overnight stay in the hospital were used. Costs of hospitalizations not due to revascularizations and emergency care visits were derived from the Dutch Costing Manual [14].

#### Costs of the Outpatient Clinic Visits

As a reference cost price of an e-visit was not available, the e-visits and in-office outpatient clinics were calculated by top-down micro-costing. In the base-case, patient-related costs were not taken into account. For the in-office outpatient clinic (ie, ECG), administrative and NP costs were taken into account. The last two were taken from the Dutch costing manual, whereas costs for the ECG were taken from the NZA list of maximum prices [14]. Hospital costs were multiplied by 1.44 (44% overhead), in accordance with the Dutch costing manual [14]. For the e-visit, administrative, video connection system, and consultation costs were taken into account. Costs of the video system were calculated by dividing the yearly subscription costs by the system's full capacity (11 patients per half-day, 110 patients a week times 50 weeks of outpatient clinic, summing up to 5500 e-visits per year). These were multiplied by 1.22 (22% overhead). A 22% overhead was assumed because of a lack of cleaning and a decrease in housing costs. The costs of an e-visit amounted to €44 (US $48), and an in-office visit cost €96 (US $106).

#### Quality-Adjusted Life Years

Utilities were derived from the Short Form Health Survey (SF-36) questionnaire [15]. These questionnaires were administered via the computer, smartphone, or tablet. Patients received an email with a URL to a web page where the SF-36 could be filled in digitally. During The Box trial, patients in both groups were asked to fill in the SF-36 three times: at 1 month, 6 months, and 12 months after inclusion. Results of the SF-36 were converted into health utilities (1=perfect health, 0= health as bad as dead) by using the established UK-based utility algorithm obtained through the University of Sheffield Licensing [16]. Multiple imputation was used to assess missing utility values. Baseline characteristics such as, but not limited to, age, gender, index event (STEMI vs NSTEMI), maximum troponin levels, and previous utilities were taken into account. Subsequently, quality-adjusted life years (QALYs) were calculated using the area under the curve method.

#### Cost-Effectiveness Acceptability Curve

Nonparametric bootstrapping was used (involving 1000 replications) to calculate uncertainty around the costs and effects estimates. Based on these results, a cost-effectiveness acceptability curve was constructed by plotting the proportion of costs and effects pairs for which the intervention is cost-effective compared to regular follow-up for a range of values of the willingness to pay for a QALY. The willingness-to-pay threshold in the Netherlands is between €20,000 (US $21,989) and €80,000 (US $87,957) per QALY [17].

#### Sensitivity Analysis of Patient-Related Costs

To analyze the potential effect of The Box on hospital and patient-related costs, a sensitivity analysis was performed. In this analysis, the costs of e-visits and in-office outpatient clinic visits were altered, as patient-related costs were included in the calculation. To calculate patient-related costs, the following costs were assumed for the in-office outpatient clinic visit: travel costs, parking costs, and 4.5 hours of loss of economic productivity multiplied by an hourly wage. For the digital outpatient clinic visit, half an hour of loss of economic productivity was assumed. The median age of the population was 59 years (IQR 53-66); therefore, it was assumed that 70% of the study population was still economically productive. The hourly wage of “economically productive” patients was €37.05 (US $40.76), whereas the hourly wage of “non-economically productive” patients (eg, retired patients) was €13.33 (US $14.66) [18]. The vast majority of the myocardial infarction population of the LUMC lives within 10 kilometers of the hospital. As such, an average distance of 7 kilometers for both groups was assumed, according to the Dutch Costing manual [14]. Parking costs were assumed to be €3.20 (US $3.52) for one hospital visit, again in accordance with the Dutch Costing manual [14]. Time for using The Box was multiplied by the hourly tariff of non-economically productive patients, as patients used this during non-office hours. A total of 10 minutes per week to take measurements was included. All costs taken into account are given in Table 1.

#### Statistical Analysis

The sample size calculation of 200 patients has been published previously. It was calculated using R statistical software version 3.2.0 for Windows (R Project for Statistical Computing). It was assumed that 95% of patients in the intervention group would have regulated BP against 75% in the control group. An α of .05, a β of .20, and a margin of 0.07 were chosen. Costs were calculated in 2020 euros and are presented as mean±SD. Differences between the intervention and control group in mean costs per category were tested for statistical significance using an independent student’s *t* test. *P* values and confidence intervals were calculated using SPSS (IBM Corp) and IBM SPSS Statistics for Windows (version 25.0; IBM Corp).

## Results

### Patient Population

In total, 200 patients (median age 59 years, 78% male) were included in the trial. These patients served as the base-case for the trial-based analysis. Of these patients, 100 (from now: 100% of the intervention group) were randomized to The Box, and 100 (from now: 100% of the control group) were randomized to the control group. In total, 12 patients (12%) in the intervention group and 8 (12%) in the control group were lost to follow-up and were not included in the base-case analysis. In both groups, 2 patients per group passed away (21% each). These patients were included in this cost-utility analysis. In total, the intervention group consisted of 88 (88%) patients and the control group of 92 (92%) patients. Results on primary and secondary outcomes have been published previously in detail [5].

### Base-Case Analysis

Mean total costs per patient were €2417±2043 (US $2657±2246) for the intervention group and €2888±2961 (US $3175±3255) for the control group. On average, costs were €471 (US $518) lower in the intervention group. This difference was, however, not statistically significant (95% CI –€275 to €1217; *P*=.22, US $–302 to $1338). Statistical significance of differences in mean costs per item per patient are given in Table 2.

**Figure 1:**
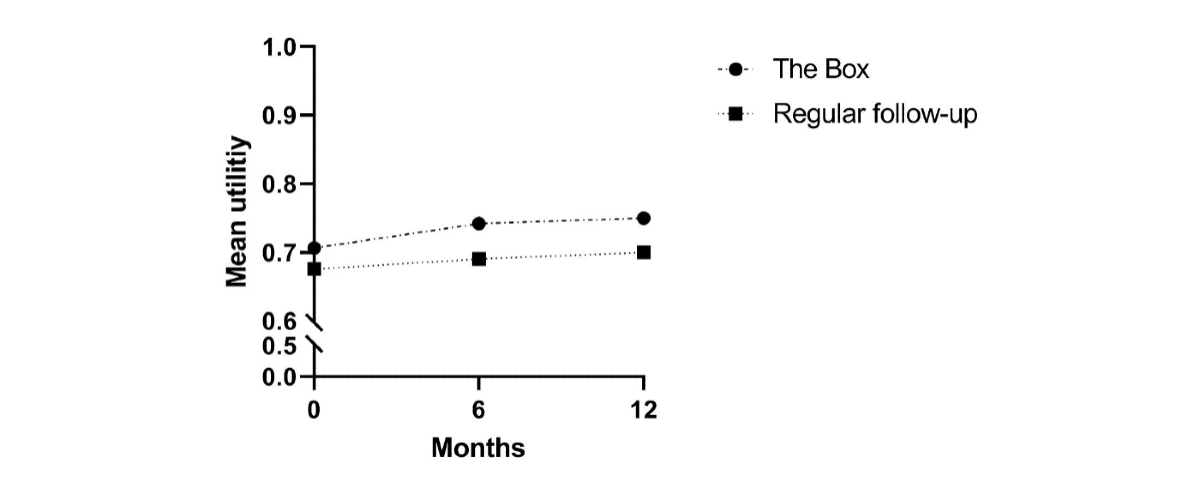
Mean pooled utilities per randomization group at one, six and twelve months after study inclusion.

Mean utilities per randomization group were noted at 1, 6, and 12 months. Differences between utilities were not statistically significant at 1 month (–0.03; 95% CI –0.07 to 0.01; *P*=.16), 6 months (–0.05; 95% CI –0.11 to 0.001; *P*=.06), and 12 months (–0.05; 95% CI –0.11 to 0.01). When converting the utilities to QALYs, the mean QALY per patient was 0.74 for the intervention group and 0.69 for the control group. This difference was statistically significant (95% CI –0.09 to –0.01; *P*=.028). Utilities are graphically represented in Figure 1.

**Table 2:** Pooled costs per patient per item for both the intervention group and regular follow-up.

Item	Intervention group, numbers	Intervention group, costs, €µ±SD	Regular follow-up, numbers	Regular follow-up, costs, €µ±SD	Difference	95% CI	*P* value
The Box	88	€318±0^a^	0	€0±0	–€318	–€318 to –€318	<.001
E-visit	148	€34±13	0	€0±0	–€34	–€36 to –€31	<.001
In-office outpatient clinic visit	181	€146±58	373	€288±46	€142	€126 to €157	<.001
Holter	89	€196±108	171	€360±84	€164	€136 to €192	<.001
Transthoracic echocardiogram	100	€151±76	178	€256±58	€105	€86 to €126	<.001
Stress echocardiogram	76	€468±204	85	€500±144	€32	–€19 to €84	.12
Emergency care visit	12	€38±104	25	€75±221	€37	–€13 to €88	.15
Hospitalization	18	€139±697	25	€186±984	€47	–€204 to €296	.72
Catheterization	13	€308±745	22	€499±946	€191	–€59 to €440	.13
Single vessel PCI^b^	10	€273±1256	4	€652±2077	€379	–€125 to €884	.14
Multivessel PCI	2	€146±963	1	€69±670	–€77	–€318 to €165	.54
Box support	1	€195±0	0	€0±0	–€195	–€195 to –€195	<.001

^a^A currency exchange rate of €1=US $1.0994 is applicable.

^b^PCI: percutaneous coronary intervention.

### Sensitivity Analysis

Mean patient-related costs were €426±114 (US $468±125) per patient in the intervention group, while mean patient-related costs in the control group were €570±92 (US $627±101). The difference of €144 (US $158) was statistically significant (95% CI €115 to €175; *P*<.001, US $127 to $193). In the sensitivity analysis, mean total costs per patient were €2842±2047 (US $3127±2252) for the intervention group and €3458±2974 (US $3805±3273) for the control group. This difference was not statistically significant (95% CI –€133 to €1365; *P*=.11, US $146 to $1365).

### Cost-Effectiveness

**Figure 2:**
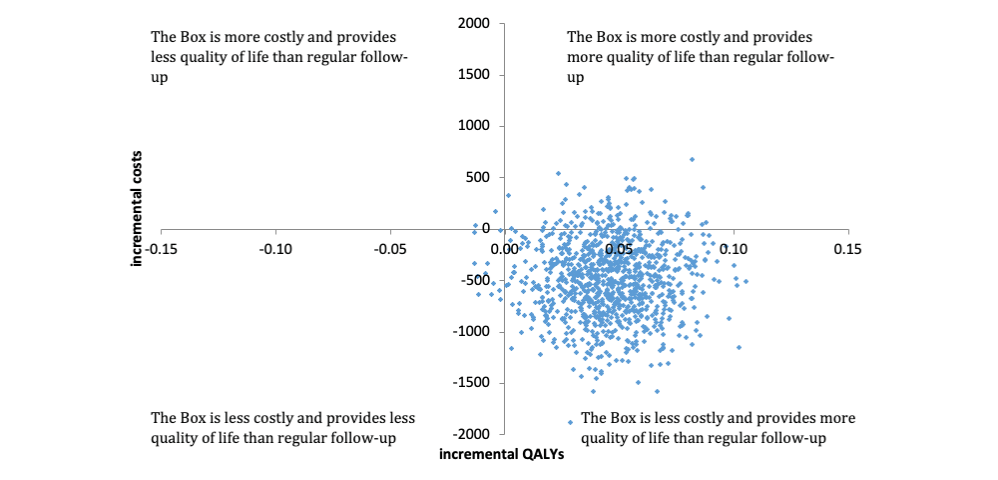
Scatter plot of incremental costs and incremental quality-adjusted life years in the base-case analysis. QALY: quality-adjusted life years.

**Figure 3:**
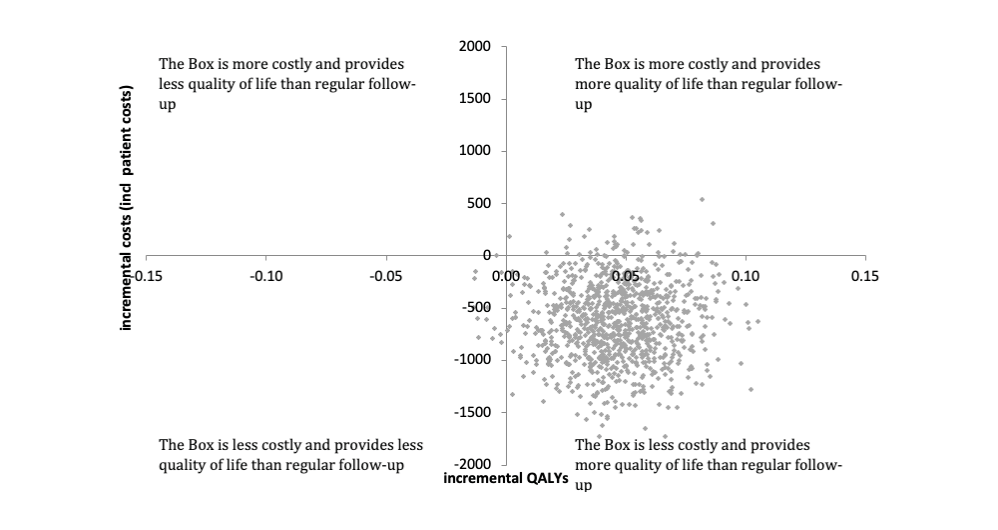
Scatter plot of incremental costs and incremental quality-adjusted life years in the sensitivity analysis. QALY: quality-adjusted life years.

**Figure 4:**
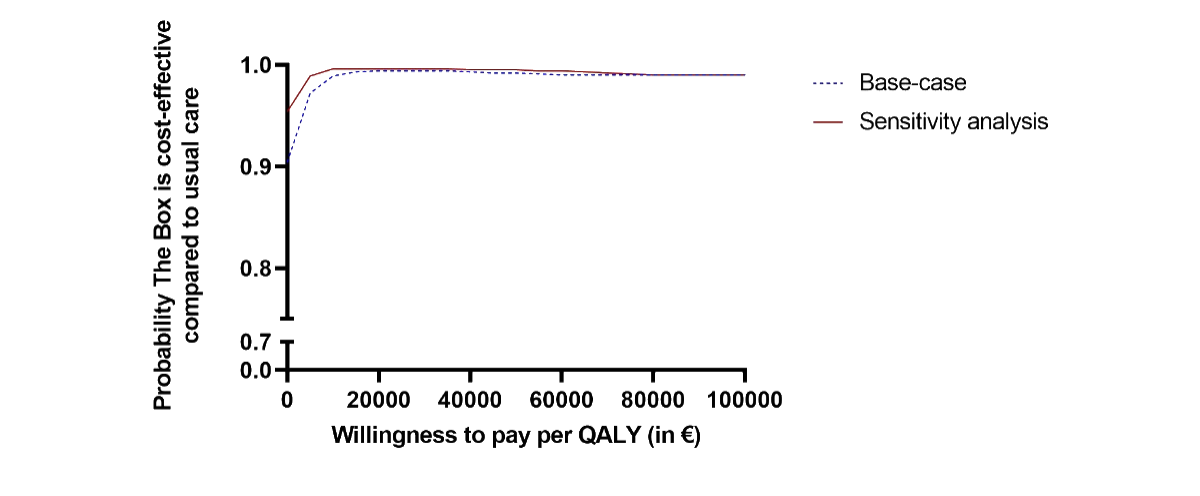
Cost-effectiveness acceptability curve. A currency exchange rate of €1=US $1.0994 is applicable. QALY: quality-adjusted life years.

Bootstrap results of the base-case and sensitivity analysis, including patient-related costs, are presented in the cost-effectiveness planes shown in Figures 2 and 3, respectively. The cost-effectiveness acceptability curves of both the base-case and the sensitivity analysis show that the probability that the Box is cost-effective compared to usual care is very high, above 0.9 for all values of the willingness to pay for a QALY (Figure 4).

## Discussion

### Principal Findings

In this paper, an RCT analyzing the cost-utility of an eHealth intervention in post-AMI patients was presented. Important findings of this paper were that, on average, costs per patient in The Box group were nonsignificant lower than in the control group and quality of life showed a small but statistically significant difference. These findings were corroborated in a sensitivity analysis.

The results from this paper can contribute to the ongoing discussions regarding telemonitoring and telerehabilitation in patients with cardiovascular disease. Rising health care costs are putting pressure on budgets for health care in all developed countries. In the Netherlands, health care costs are an estimated 11% of the total gross domestic product (GDP). Costs are growing faster than the economy. Without a significant change in the way health care is delivered by 2040, it is expected that 30% of the GDP will be spent on health care [19]. This increase in costs has been attributed to increased volumes, patients with multi-morbidity, as well as the use of more sophisticated (and therefore expensive) clinical technology [20]. eHealth has been identified as a tool to lower costs while at the same time increasing quality by focusing more on preventing disease (instead of treating). Moreover, it could reduce costs by helping to integrate care by easing communication between care providers and reducing duplication of diagnostic testing. Lastly, it could reduce costs as patients are enabled to perform some of their diagnostic tests by themselves instead of by trained health care staff [21]. Although these are rather general remarks, the results of this study support some of this theory. In this study, patients were able to measure their own BP, ECG, and weight and transfer it to the hospital. This enabled the replacement of two in-office outpatient clinic visits with two digital outpatient clinic visits, with consequently cost reductions, as the price of an e-visit is about half the price of an in-office outpatient clinic. Potentially, with 34,000 AMI patients in the Netherlands per year, the eHealth intervention could save an estimated €16.1 million euros (US $17.7 million US dollars) in health care costs for cardiology departments annually [18,22].

### External Validity

This RCT was performed in Dutch patients who suffered from AMI. In the Netherlands, distances are known to be small. The average distance between the hospital and the patient’s home was 7 kilometers [14]. Moreover, in this study, it was estimated that 30% of patients were retired. In a population, however, where more patients are still working and distances are larger, cost savings due to eHealth might be higher. A sensitivity analysis, taking into account the patient-related costs of an e-visit, demonstrated that cost savings of The Box could be higher. The costs of devices of The Box should be incorporated as well. These costs contribute significantly to the total costs of the intervention group. In larger populations, a cost reduction could be achieved by reducing the price per device due to larger volumes. A reduction in costs for The Box could result in a statistically significant reduction in total costs per patient compared to the control group. It could be expected that in such a scenario, the cost reduction in the intervention group could reach statistical significance. Moreover, further selection of subpopulations that are most likely to benefit from The Box could improve cost-effectiveness as well.

### Literature

To our knowledge, this is the first paper to evaluate the cost-utility of remote monitoring compared to regular follow-up in the outpatient care setting of post-AMI patients. eHealth is a rather broad term, encompassing almost all use of information technologies in health care. It is a relatively new concept. Few RCTs have been performed. A recent systematic review found 16 cost evaluations of RCTs in eHealth, ranging from internet-based cognitive behavioral therapy for depression to telemonitoring for patients with congestive heart failure [23]. As these patient populations and interventions differ from our patient population and intervention, comparing the results is difficult. Previous studies mainly found eHealth to be cost-effective but predominantly leading to an increase in costs. Our study found a (nonsignificant) reduction in costs, which is very likely to be due to the design of this study; eHealth was used to partially replace regular care, while in most eHealth studies, it is provided on top of regular care. One study evaluated telerehabilitation in post-AMI patients [24] and found the intervention to be cost-saving. A Dutch study with a comparable design and patient population corroborated these findings [25]. However, although this study evaluated to some extent a comparable patient population, a different intervention was performed. The intervention involved a telerehabilitation program and focused on exercise. Digital outpatient clinic visits were not part of the intervention [24]. These factors could explain the differences in the cost reductions found in both studies.

### Limitations

For the interpretation of the results, some limitations have to be taken into account. For the trial-based analysis, only data from the Department of Cardiology of the LUMC were used.

Secondly, this study was performed in a tertiary care center by a project dedicated team. It is therefore unknown if the percentages found in this trial will be similar in other medical centers, where care for post-AMI patients is delivered by cardiologists, and a project dedicated team is unfeasible due to lower volumes and other financing structures. Thirdly, this cost-utility calculation was done from a department’s perspective. Costs generated in other departments were not taken into account. Therefore, total costs could be underestimated. However, as it is assumed this is equally distributed, it has most likely a limited effect on the difference between the intervention and control group. In the base-case analysis, furthermore, patient-related costs were not taken into account as well. This might have led to an underestimation of costs in the control group, as patient-related costs are assumed to be higher. The sensitivity analysis indicated that if patient-related costs are included, The Box is likely to be more cost-effective. Fourthly, to convert SF-36 scores into utilities, the UK algorithm was used. As such, as there might be subtle differences between the UK and Dutch patient populations in basic SF-36 scores, this might have skewed the utility data slightly. Nevertheless, as the algorithm was used for both the intervention group and control group, there is no reason to believe another algorithm would have changed the conclusion that is based on the utility data.

### Conclusions

The most important conclusion is that remote monitoring in post-AMI patients is likely to be cost-effective compared to usual care (and at least not more expensive). This intervention in the outpatient care setting of post-AMI patients could be a valuable additive in restraining rising health care costs or in situations where physical outpatient clinic visits are undesirable.

## Abbreviations

AMI: acute myocardial infarction
BP: blood pressure
CCU: cardiac care unit
ECG: electrocardiogram
ESC: European Society of Cardiology
GDP: gross domestic product
LUMC: Leiden University Medical Center
NP: nurse practitioner
NSTEMI: non-ST-elevation myocardial infarction
NZA: Nederlandse Zorgautoriteit
PCI: percutaneous coronary intervention
QALY: quality-adjusted life years
RCT: randomized controlled trial
SF-36: Short Form Health Survey-36
STEMI: ST-elevation myocardial infarction
TTE: transthoracic echocardiogram

## Data Availability

The data underlying this article will be shared following a reasonable request to the corresponding author.
